# A DFT and Semiempirical Model-Based Study of Opioid Receptor Affinity and Selectivity in a Group of Molecules with a Morphine Structural Core

**DOI:** 10.1155/2012/682495

**Published:** 2012-12-13

**Authors:** Tamara Bruna-Larenas, Juan S. Gómez-Jeria

**Affiliations:** Department of Chemistry, Faculty of Sciences, University of Chile, P.O. Box 653, Santiago, Chile

## Abstract

We report the results of a search for model-based relationships between mu, delta, and kappa opioid receptor binding affinity and molecular structure for a group of molecules having in common a morphine structural core. The wave functions and local reactivity indices were obtained at the ZINDO/1 and B3LYP/6-31G^∗∗^ levels of theory for comparison. New developments in the expression for the drug-receptor interaction energy expression allowed several local atomic reactivity indices to be included, such as local electronic chemical potential, local hardness, and local electrophilicity. These indices, together with a new proposal for the ordering of the independent variables, were incorporated in the statistical study. We found and discussed several statistically significant relationships for mu, delta, and kappa opioid receptor binding affinity at both levels of theory. Some of the new local reactivity indices incorporated in the theory appear in several equations for the first time in the history of model-based equations. Interaction pharmacophores were generated for mu, delta, and kappa receptors. We discuss possible differences regulating binding and selectivity in opioid receptor subtypes. This study, contrarily to the statistically backed ones, is able to provide a microscopic insight of the mechanisms involved in the binding process.

## 1. Introduction

 Molecular recognition processes control a huge number of aspects of life on Earth. The ability of molecules to recognize a certain pattern of atom distribution and not another is central to catalysis, drug effects, chemical reactivity, and so forth. Concerning the recognition by a drug of one or more receptors, this is a phenomenon that still needs to be fully understood to design new agonists or antagonists for a given receptor type. The central problem of the drug-receptor interaction is the following: how can a certain molecule be recognized by two or more receptors and display different affinities for them? Among the molecules having this interesting property we may cite dopaminergic, serotoninergic, and opioid compounds. In the following we shall focus on the latter.

Regarding opioids there is abundant evidence for the existence of four major classes of receptors in the central nervous system (CNS), designated as *μ*, *δ*, *κ*, and nociceptin, as well as subtypes within the first three classes (we employed capital letters to avoid confusions because similar small Greek letters are used to design reactivity indices used below). Each receptor type has a distinct selectivity profile and a unique distribution within the CNS. They are activated both by endogenously produced opioid peptides and by exogenously administered opiate compounds, some of which are not only among the most effective analgesics known but also highly addictive and abused (they are not the only ones to be activated by exogenous compounds; serotoninergic 5-HT_2_ receptors are activated by hallucinogens like LSD; e.g.).

The great medical importance of these and similar molecular systems requires research on their quantitative structure-activity relationships (QSAR) in order to improve our knowledge about how receptor binding, selectivity, and pharmacological effects are achieved. The set of molecular features which are necessary for binding are summarized in the “interaction pharmacophore” and those necessary for pharmacological effects (agonism, antagonism, etc.) in the “agonist pharmacophore,” “antagonist pharmacophore”, and so forth. In the case of opiates there are several lines of research dealing with the synthesis and pharmacological evaluation of derivatives of endogenous opioid peptides (enkephalins, which are *δ* receptor ligands, *β*-endorphin, which binds to all opioid receptors, and dynorphins, which exert their effects primarily through the *κ*-opioid receptor). Another line of research does the same but with rigid opiates derived from morphine, pethidine, naltrexone, and other exogenous molecules. Finally, there have been some efforts to combine experimental results with crystallographic data and quantum chemical calculations into several pharmacophore models.

Regarding the last line of research, Gorin and Marshall defined a model for the opiate receptor by using a computer-based molecular display, and X-ray crystallographic input data. The model can explain the stereochemistry of the way in which the morphine, morphinan, and oripavine classes of compounds interact with the receptor. The minimal structural unit of the enkephalins demonstrated to be pharmacologically active, Tyr-lyGly-Phe, was also fitted to this model by using a systematic search of conformational space. This model for the analgesic pharmacophore utilizes the previously recognized requirement of the phenolic ring and tertiary amine of morphine. To be consistent with the stereospecific activity of the morphine, morphinan, and oripavine classes of compounds, it was also proposed that atoms C5 and C6 of the C ring of morphine are an additional requirement [1]. Burt et al. have identified molecular features and types of receptor interactions that modulate agonist/antagonist potencies in diverse classes of opiates. Using the fused-ring opiates as a template for the interaction of opiates at the receptor site, they have developed hypotheses that can explain pure agonist, pure antagonist, and mixed agonist/antagonist activity not only for fused-ring opiates, but also for peptide opiates and flexible opiates such as the 4-phenylpiperidines and 3-phenylpiperidines [2]. Fournie-Zaluski et al. suggested that *μ* receptors bind preferentially to highly hydrophobic compounds with compact structures while *δ* receptors exhibit a stronger affinity for larger peptides with hydrophilic components. For this they used a mixture of experimentally measured IC_50_ values and opioid activities [3]. Loew et al. examined the conformational behavior of four tetrapeptide enkephalin analogues (Tyr-Gly-Gly- Phe-OH, Tyr-Gly-Gly-Phe-NH_2_, Tyr-D-Ala-Gly-Phe-NH_2_, and Tyr-D-Ala-Gly- (NMe)Phe-NH_2_) to identify conformations that are active and inactive at the opiate analgesic receptor. Thus, on the basis of conformational data, the Tyr-Gly-Gly-Phe-NH_2_ analogue is predicted to have very weak opiate activity [4]. Lavecchia et al. modeled the *κ*-opioid receptor-agonists interactions using pharmacophore-based and docking simulations [5]. Their data provide additional evidence that *δ*-opioid agonists and antagonists interact within the same ligand-binding domain in opioid receptors and that hydrophobic substituents at the C-terminus of the Dmt-Tic pharmacophore augment *μ*-opioid receptor affinity. Thus, Dmt-Tic (Dmt = 2′,6′-dimethyl-L-tyrosine, Tic = 1,2,3,4-tetrahydroisoquinoline-3-carboxylic acid) with hydrophobic C-terminal substituents enhanced *μ* affinity to provide *δ* antagonists with dual receptor affinities and bifunctional activity. Bonner et al. prepared topographically constrained analogues of the highly *μ*-opioid-receptor-selective antagonist CTAP (H-D-Phe-c [Cys-Tyr-D-Trp-Arg-Thr-Pen]-Thr-NH_2_) [6]. Molecular modeling based on 2D NMR revealed that low energy conformers of peptides with similar biological activities had similar aromatic pharmacophore orientations and interaromatic distances. Peptides that exhibit *μ* antagonism have interaromatic distances of 7.0–7.9 Å and have their amino terminal aromatic moiety pointing in a direction opposite to the orientation of the amino terminus. Peptides with *δ* opioid activity displayed an interaromatic distance of <7 Å and had their amino terminal aromatic moiety pointing in the same direction as the amino terminus. Bernard et al. extended the conformationally sampled pharmacophore (CSP) method to peptide ligands using replica exchange molecular dynamics simulation for conformational sampling [7]. The developed 2D CSP indicates that the spatial relationship of the basic nitrogen and the hydrophobic moiety in the *δ* opioid ligands differentiates activity. Such an overlap is expected because all the ligands bind to the same receptor and support a model where both classes of ligands interact with the *δ* receptor via the same binding mode. However, there exist high-probability regions that are primarily sampled by agonists versus antagonists and vice versa for both the peptide and nonpeptide ligands. Kuz'mina et al. presented in a series of papers a general model of the opiate pharmacophore [8–10]. Bernard et al. extended the CSP method to obtain quantitative models of *δ* opioid ligand efficacy and affinity [11]. The models obtained for a structurally diverse set of peptide and nonpeptide *δ* opioid ligands offer good predictions with *R*2 values > 0.9, and the predicted efficacy for a set of test compounds was consistent with the experimental values. Later Shim et al. applied the CSP method to develop a predictive model of the efficacy of *μ*-opioid receptor ligands [12]. Their model predicts (1) that interactions of ligands with the B site, as with the 19-alkyl substituents of oripavines, modulate the extent of agonism; (2) that agonists with long N-substituents, as with fentanyl and N-phenethylnormorphine, can bind in an orientation such that the N substituent interacts with the B site that also allows the basic N-receptor Asp interaction essential for agonism; (3) that the *μ* agonist herkinorin, that lacks a basic nitrogen, binds to the receptor in a manner similar to the traditional opioids via interactions mediated by water or an ion.

 On the experimental side, biological studies of endogenous opioid peptides, as well as synthetic analogues, have led to the hypothesis that the tetrapeptide sequence from Tyr^1^ to Phe^4^ is an important requirement for activity. It was proposed that the N-terminal tetrapeptide sequence of endogenous peptides carries the “message”, which is responsible for mediating the opioid effect. The C-terminal segments of these peptides, which differ in length and physical-chemical character, play an “address” role in conferring selectivity for different opioid receptor types. Lipkowski et al. synthesized analogues of leucine-enkephalin and dynorphin(1–8) in which the N-terminal dipeptide message sequence has been replaced by oxymorphone or naltrexone (these molecules are called Alkaloid-Peptide Hybrids) [13]. Their results suggest that the selectivity for different opioid receptor types depends on a balance between the affinities of the message and address components. In cases where these components have comparable receptor affinities, the address can significantly shift selectivity by increasing affinity for one receptor type while reducing affinity for other types. When the message component has high affinity for a particular receptor type, the modulatory role of the address is expressed mainly by reducing the affinity of the ligand for other opioid receptor types. Portoghese et al. also synthesized bivalent ligands consisting of oxymorphamine and [D-G1u^2^]enkephalin pharmacophores linked through a spacer attached to the 6-amino group of the former and D-Glu of the latter to investigate the possible coexistence of *μ* and *δ* recognition sites in the same opioid receptor complex [14]. They suggest that the results are consistent with the simultaneous occupation of *μ* and *δ* by a single bivalent ligand, but they are also in harmony with the interaction of the bivalent ligands with an opioid receptor and an accessory binding site. In another paper Portoghese et al. investigated whether one or two pharmacophores are required for the *κ* opioid receptor selectivity of the bivalent opioid antagonist norbinaltorphimine [15]. They suggested that the *κ* selectivity of this kind of molecules is derived from the portions of the second halves of these molecules in that they mimic key address components of dynorphin at *κ* opioid receptors. Larson et al. also examined the effect of structural modifications on the affinity of norBNI analogues for wild-type and mutant *κ* and *μ* opioid receptors expressed in COS-7 cells [16]. It is suggested that the antagonist pharmacophore is bound within this highly conserved region of the *κ* or mutant *μ* receptor and that an anionic residue at the top of transmembrane helix 6 provides additional binding affinity. Lazarus et al. prepared analogues of the Dmt-Tic pharmacophore to test the hypothesis that a spacer and a third aromatic center in opioid peptides are required to convert a *δ*-antagonist into ligands with *δ*-agonist or with mixed *δ*-antagonist/*μ*-agonist properties [17, 18]. These data confirm that the distance between the Dmt-Tic pharmacophore and a third aromatic nucleus is an important criterion in converting Dmt-Tic from a highly potent *δ*-antagonist into a potent *δ*-agonist or into ligands with mixed *δ*- and *μ*-opioid properties. Grundt et al. start from the fact that the trans-(3,4)-dimethyl-4-(3-hydroxyphenyl)piperidines are a unique class of opioid antagonists that have recently provided selective antagonists for *μ*-opioid receptors and *κ*-opioid receptors. Molecular modeling indicated a strong structural similarity between the parent of this series and 2-amino-1,1-dimethyl-7-hydroxytetralin. Introduction of a methoxy group in the 3-position increased potency at *μ* and *κ* receptors, suggesting that this aminotetralin skeleton can be utilized as a new scaffold for the design of selective opioid receptor antagonists [19]. Balboni et al. developed a series of 17 analogues on the basis of the general formula H-Dmt-Tic-NH-∗CH(R)-R′ (∗ denotes chirality; R charged, neutral, or aromatic functional group; R′ = –OH or –NH_2_) [20]. Thus, these C-terminally extended analogues indicated that an amino acid residue containing a single charge, amino or guanidino functionality, or aromatic group, substantially altered the *δ*-opioid receptor activity profile (selectivity and antagonism) of the Dmt-Tic pharmacophore, which suggests that the C-terminal constituent plays a major role in determining opioid receptor activity as an address domain. Daniels et al. synthesized and evaluated bivalent ligands (KDAN series) containing *δ*-antagonist (naltrindole) and *κ*_1_-agonist (ICI-199,441) pharmacophores [21]. The data suggested that KDAN-18 bridges phenotypic *δ*_2_- and *κ*_1_- receptors. They presented a conceptual model to explain the organizational differences between the opioid receptors that give rise to the phenotypes (*δ*_1_, *δ*_2_, *κ*_1_, *κ*_2_). Peng et al. synthesized a series of homo- and heterodimeric ligands containing *κ* agonist and *μ* agonist/antagonist pharmacophores joined by a linker chain of varying lengths. They were evaluated in vitro for their binding affinity at *μ*, *δ*, and *κ* opioid receptors. The functional activities of these compounds were measured in the [35S]-GTP*γ*S binding assay [22]. The data suggest that the stereochemistry of the pharmacophores, the N-substituents of the pharmacophore, ester linkages, and the spacer length were crucial factors for optimal interactions of such ligands at opioid receptor binding sites. Agnes et al. designed a single peptide which can interact with *δ* and *μ* opioid receptors as agonists and with CCK receptors as antagonists [23]. These results provide evidence supporting the concept that opioid and CCK receptors have overlapping pharmacophores required for binding affinity and biological activity and that design of overlapping pharmacophores of two peptides into a single peptide is a valid approach. Balboni et al. analyzed the substitution of Gly with side-chain-protected or unprotected Lys in lead compounds containing the opioid pharmacophore Dmt-Tic [H-Dmt-Tic-Gly-NH-CH_2_-Ph, *μ* agonist/*δ* antagonist; H-Dmt-Tic-Gly-NH-Ph, *μ* agonist/*δ* agonist; and H-Dmt-Tic-NH-CH_2_-Bid, *δ* agonist (where Bid = 1H-benzimidazol-2-yl)] obtaining a new series of compounds endowed with distinct pharmacological activities [24]. The presence of a Lys linker provides new lead compounds in the formation of opioid peptidomimetics containing the Dmt-Tic pharmacophore with distinct agonist and/or antagonist properties. The change of biological activity, receptor binding, and selectivity upon changes in the molecular structure have been analyzed in several recent publications [25–28].

 The abovementioned experimental work suggests very interesting and challenging hypotheses about opiate receptor binding and selectivity that need to be explained at the microscopic level. In our Laboratory, and using a model-based method, we have addressed this question through the analysis of the different modes of binding of molecules to *μ*, *δ*, and *κ* opiate receptors in order to generate a binding pharmacophore [29–31].

 As, on the one hand, the model-based method has been perfected by the formal addition of new terms having definite chemical meaning and, on the other hand, we have written new computer codes to extract new and useful information from standard quantum chemical packages, we now have newer tools that are able to detect the microscopic factors regulating affinity and selectivity. This paper uses quantum chemical methods in an attempt to advance the knowledge of the relationship between electronic structure and the binding to *μ*, *δ*, and *κ* receptors in a series of high affinity opioid receptor ligands, whereby the phenolic OH group of nalbuphine, naltrexone methiodide, 6-desoxonaltrexone, hydromorphone, and naltrindole was replaced by a carboxamido group and the furan ring was opened to the corresponding 4-OH derivatives to the receptor.

## 2. Methods, Models, and Calculations

 It was during the second half of the 1950 decade when the first applications of Molecular Orbital (MO) theory to the study of questions of pharmacological interest began to appear in the scientific literature [32–35]. Quantum chemistry provided the Linear Combination of Atomic Orbital approximation (LCAO) [36] and the work of Fukui et al. about reactivity and weak molecular interactions [37–39]. At that time progress was slow due to the lack of a reasonable theoretical framework within which to work and to the complexity and long calculation time of the electronic structures. This last barrier was overcome with the availability of faster and faster computers (calculations lasting about 24 hours or more in the 1960s are done today in a matter of seconds or minutes). This, together with the new all-valence electron semiempirical [40], and the *ab initio *Hartree-Fock (HF) [41] and Density Functional (DFT) [42] methods finally placed biologically important substances in the realm of feasible MO studies.

 If compounds in a congeneric or a homologous series of compounds differ only by one substituent, it is not unreasonable to assume that the difference in their binding to a receptor site is due solely to differing properties of the substituents. Since there is no *a priori* or *a posteriori* knowledge of the particular atoms primarily responsible for the activity of a drug, we will require in principle that each atom of the drug interact with each atom of the drug receptor site. In such a case, if the model works, that is, can represent the data, it may give new insights into the mechanism of binding. We must note that the assumption implicit in this treatment is that receptor structure and mechanism of action remain constant while only the binding (affinity) varies throughout the series. In 1967 Klopman and Hudson published a general perturbation treatment of chemical reactivity, not restricted to *π*-conjugated molecules, in which allowance is made for ionic interactions [43–45]. As this theory represents the interaction energy in terms of atom-atom interactions it was only a question of time before the first papers applying it to the study of the activity of biological molecules appeared [46–48]. Given that, as far as we know, the last paper not belonging to our group, and using the model we are using here, was published in 1979 [49], we shall present in the following a detailed description of this model. We are doing so to distinguish it very clearly from the statistics-backed methodologies.

 Let us consider the state of thermodynamic equilibrium, and a 1 : 1 stoichiometry in the formation of the drug-receptor complex:

(1) $$\begin{array}{c} D_{i}+R⇌D_{i}R, \end{array}$$

where *D*_*i*_ is the drug, *R* is the receptor, and *D*_*i*_ *R* is the drug-receptor complex. According to statistical thermodynamics the equilibrium constant, *K*_*i*_, is expressed as [50]

(2) $$\begin{array}{c} K_{i}=\frac{Q_{D_{i}R}}{Q_{D_{i}}Q_{R}}\exp\left(-\frac{\Delta \varepsilon_{0}^{i}}{kT}\right), \end{array}$$

where Δ*ε*_0_^*i*^ is the difference between the ground-state energy of *D*_*i*_ *R* and the energies of the ground states of *D*_*i*_ and *R*:

(3) $$\begin{array}{c} \Delta \varepsilon_{0}^{i}=\varepsilon_{D_{i}R}-\left(\varepsilon_{D_{i}}+\varepsilon_{R}\right)\; \end{array}$$

and the Q's are the total partition functions (PF) measured from the ground state (in solution). *T* and *k* are the temperature and the Boltzmann constant, respectively. First of all, if we consider that for almost all polyatomic molecules the Boltzmann factors of the excited electronic states are negligible compared to those of the ground state, we may consider only the electronic ground state in the PF. Second, we shall consider that the rotational and vibrational motions are independent and uncoupled and that at body temperature, the vibrational PFs have a value close to 1 [51]. Third, we shall employ the classical expression for the rotational PF together with the assumption that the rotational PFs of the receptor and the drug receptor are similar (this requires that the receptor molecule be much greater than the drug molecule). In logarithmic form, (2) transforms into [52]

(4) $$\begin{array}{c} \log\;K_{i}=a+bM_{D_{i}}+c\log\left[{\left(\frac{\sigma_{D_{i}}}{\text{ABC}}\right)}^{1/2}\right]+d\Delta \varepsilon_{i}, \end{array}$$

where *a*, *b*, *c*, and *d* are constants, *M* is the drug's mass, *σ* its symmetry number, and ABC the product of the drug's moment of inertia about the three principal axes of rotation.

 The interaction energy, Δ*ε*_*i*_, cannot be determined directly, either due to the size of the receptor or to the lack of knowledge of its molecular structure. Nevertheless, as we are dealing with a weak drug-receptor interaction, we can employ Perturbation Theory in the Klopman-Hudson form to evaluate Δ*ε*_*i*_. According to this method, the change in electron energy, Δ*E*, associated with the interaction of atoms *i* and *j* is

(5) ΔE=∑p[QiQjRij+(12)×(βij2)∑m ∑nDmiDn′j(Em−En′)(12)−(βij2)∑m ∑´nDm′iDnj(Em′−En)  ],

where *Q*_*i*_ is the net charge of atom *i, D*_*mi*_ is the orbital charge of atom *i* in the MO *m*, *β*_*ij*_ is the resonance integral, and *E*_*m*_ (*E*_*m*′_) is the energy of the *m*-th (*m*′-th) occupied (virtual) MO of the drug, with *n* and *n*′ standing for the receptor. The value of *β*_*ij*_ is kept independent of the kind of AO because the drug-receptor complex does not involve covalent bonds. The summation on *p* is over all pairs of interacting atoms of the drug and the receptor.

 The first term of the right side of (5) represents the electrostatic interaction between two atoms having net charges *Q*_*i*_ and *Q*_*j*_. The second and third terms introduce the interactions between the occupied and empty MOs of the drug and those of the receptor. 

As the MO energies of the receptor are not known, in the first applications of this method these values were replaced by constants [48, 49]. We followed a different approach. Noting that 1/(*E*_*m*_ − *E*_*n*′_) and similar terms can be written in the form 1/(1 − *x*), we may expand them as a convergent infinite series. After a little algebra we obtain [53]

(6) ΔE=a+∑i[eiQi+fiSiE+siSiN]+∑i∑m[hi(m)Fi(m)+ji(m)SiE(m)]+∑i∑m′[ri(m′)Fi(m′)+ti(m′)SiN(m′)]+Φ,

where *a*, *e*, *f*, *g*, *h*, *j*, *r*, and *t* are constants and the summation on *i* is now only over the drug's atoms. *S*_*i*_^*E*^ and *S*_*i*_^*N*^ are, respectively, the total atomic electrophilic and nucleophilic superdelocalizabilities of Fukui et al. [54]. *F*_*i*,*m*_ is the Fukui index of atom *i* in occupied (empty) MO *m* (*m*′). The total atomic electrophilic superdelocalizability (ESD) of atom *i* is defined as

(7) $$\begin{array}{c} S_{i}^{E}=\sum_{m}\frac{F_{i,m}}{E_{m}}=\sum_{m}S_{i}^{E}\left(m\right), \end{array}$$

where the summation on m runs only over the occupied MOs. *S*_*i*_^*E*^(*m*) is called the orbital electrophilic superdelocalizability of atom *i* at MO *m*. 

 The total atomic nucleophilic superdelocalizability (NSD) of atom *i* is defined as

(8) $$\begin{array}{c} S_{i}^{N}=\sum_{m\prime }\frac{F_{i,m\prime }}{E_{m\prime }}=\sum_{m^{\prime }}S_{i}^{N}\left(m^{\prime }\right), \end{array}$$

where the summation on *m*′ runs only over the empty MOs. *S*_*i*_^*N*^(*m*′) is called the orbital nucleophilic superdelocalizability of atom *i* at MO *m*′. Φ stands for the remaining series expansion terms. The summation on *p* (i.e., the atoms involved in the interaction) is hidden for the sake of clarity. *S*_*i*_^*E*^ is associated with the total electron-donating capacity of atom *i* and *S*_*i*_^*N*^ with its total electron-accepting capacity. These indices are very useful to compare the reactivity of a similar atomic position through a series of molecules because they incorporate the eigenvalue spectrum which is usually different in each molecular system. The orbital components, *S*_*i*_^*E*^(*m*) and *S*_*i*_^*N*^(*m*′), become important when fine aspects of the drug-receptor interaction are needed for a more complete explanation.

*The most important feature of* (6)* is that it includes terms belonging only to the drug molecule*. During the year 2011 we analyzed several terms composing Φ and, after long and tedious algebra, we found that we may associate some of them with a set of local atomic reactivity indices recently proposed by one of us [55]. These indices are defined within the Hartree-Fock LCAO-MO framework as follow.

 The local atomic electronic chemical potential of atom *i*, *μi*:

(9) $$\begin{array}{c} \mu_{i}=\frac{E_{\text{oc}}^{*}-E_{\text{em}}^{*}}{2}, \end{array}$$

where *E*_oc_* is the upper occupied MO with a nonzero Fukui index and *E*_em_* is the lowest empty MO with a non-zero Fukui index.

 The local atomic hardness of atom *i*, *η*_*i*_:

(10) $$\begin{array}{c} \eta_{i}=E_{\text{em}}^{*}-E_{\text{oc}}^{*}. \end{array}$$

The local atomic softness of atom *i*, *ς*_*i*_, defined as the inverse of the local atomic hardness.

 The local electrophilic index of atom *i*, *ω*_*i*_:

(11) $$\begin{array}{c} \omega_{i}=\frac{\mu_{i}^{2}}{2\eta_{i}}. \end{array}$$

The maximal amount of electronic charge that an electrophile may accept, *Q*_*i*_^max^:

(12) $$\begin{array}{c} Q_{i}^{\max}=\frac{-\mu_{i}}{\eta }. \end{array}$$

What is the relationship between these local atomic indices and the global indices obtained within Density Functional Theory? [56–70]. Let us analyze a couple of examples. DFT defines the global (molecular) electronic chemical potential, *μ*, and the global hardness, *η*, as (Koopman's theorem was used)

(13) $$\begin{array}{c} \mu =\frac{E_{\text{HOMO}}+E_{\text{LUMO}}}{2}, \end{array}$$

(14) $$\begin{array}{c} \eta =E_{\text{LUMO}}-E_{\text{HOMO}}. \end{array}$$

Here, *E*_HOMO_ and *E*_LUMO_ are, respectively, the eigenvalues of the higher occupied and lowest unoccupied MOs of the molecule. DFT chemical potential measures the escaping tendency of electrons from a system, so electrons flow from regions with higher chemical potential to areas with lower chemical potential until *μ* becomes uniform throughout [70]. Global hardness is just the HOMO-LUMO gap and can be interpreted as the resistance of the chemical potential to change in the number of electrons.

 In the case of the local atomic electronic chemical potential *μ*_*i*_ (9), *E*_occ_* is the eigenvalue of the highest occupied MO that has a non-zero electron population on atom *i*. As Molecular Orbitals of large molecules are not localized over all the system, the value of *μ*_*i*_ will not be the same for all the atoms. The same definition holds for the local atomic hardness, the local atomic softness, the local electrophilic index, and the maximal amount of electronic charge that an electrophile may accept (10)–(12).

For this reason these local atomic indices are the local atomic analogues of similar global reactivity indices currently used in today's quantum chemistry [56–70] and can be interpreted in a similar way. The general meaning of these local atomic indices is one *μ*_*i*_ which is a measure of the tendency of an atom to gain or lose electrons; a large negative value indicates a good electron acceptor atom while a small negative value implies a good electron donor atom. The local atomic hardness can be interpreted as the resistance of an atom to exchange electrons with the environment. The local electrophilic index is associated with the electrophilic power and includes the tendency of the electrophile atom to receive extra electronic charge together with its resistance to exchange charge with the medium. This index can be viewed as a measure of the electrophilicity power because it is an analogous of the classical electrostatics power, *V*2/*R*, and *μ* and *η* serve the purpose of potential (*V*) and resistance (*R*), respectively [70].

 The insertion of (6) and (9) to (12) into (4) leads to the following final equation:

(15) log Ki=a+bMDi+clog[(σDiABC)1/2]+∑j[ejQj+fjSjE+sjSjN]+∑j ∑m[hj(m)Fj(m)+xj(m)SjE(m)]+∑j ∑m′[rj(m′)Fj(m′)+tj(m′)SjN(m′)]+∑j[gjμj+kjηj+ojωj+zjςj+wjQjmax].

Then, for *N* (*i* = 1, *N*) molecules we have a set of simultaneous equations 15. In principle, this system of simultaneous equations holds for the atoms *j* of the molecule directly perturbed by their interaction with the receptor. Combined with the usual multiple-regression techniques, these equations can be usefully applied to estimate the relative variation of log *K*_*i*_ in the family of molecules analyzed. Also, they can be used to determine which atoms are directly concerned in the formation of the drug-receptor complex. Here statistical analysis is used, *not to see whether there is a structure-activity relationship, but to find the best one*.

 On the other hand, the moment of inertia term was analyzed and it was possible to show that it can be expressed in a first approximation as [71–73]

(16) $$\begin{array}{c} \log\left[{\left(\text{ABC}\right)}^{-1/2}\right]=\sum_{t}\sum_{t}m_{i,t}R_{i,t}^{2}=\sum_{t}O_{t}, \end{array}$$

where the summation over *t* is over the different substituents of the molecule, and *m*_*i*,*t*_ is the mass of the *i*th atom belonging to the *t*-th substituent, *R*_*i*,*t*_ being its distance to the atom to which the substituent is attached. This approximation allows us to transform a molecular property into a sum of substituent properties. As the physical interpretation of these terms it was proposed that they represent the fraction of molecules attaining the proper orientation to interact with the receptor. We have called them Orientation Parameters.

 The success of this method was appreciated when it was applied to a great variety of systems: molecules interacting with serotoninergic receptors [74–78], opiates interacting with opioid receptors [29–31], carbamate insecticides [72], molecules interacting with dopaminergic receptors [79], kynurenic acid derivatives interacting with Gly/NMDA sites [80], cannabinoid derivatives interacting with the CB1 and CB2 receptors [81, 82], the inhibition of wild-type and drug-resistant HTV-1 reverse transcriptase by some thiazolidenebenzenesulfonamide derivatives [83], and the interaction of some *N*,*N*-dialkyl-2-phenylindol-3-ylglyoxylamide derivatives with the peripheral benzodiazepine receptor [84]. Two results stress the goodness of this model. The first one was the successful prediction of the hallucinogenic activity of (±)-1-(2,5-dimethoxy-4-nitrophenyl)-2-aminopropane and the approximate dose humans could take [78]. The second one was when we could not obtain a statistically significant equation for the dopaminergic *D*_2_ binding affinity of some apomorphines. We were able to show that the experimental results reported were wrong [79]. Recent results (accepted for publication) were obtained for relationships between accumulation data and molecular structure in a group of pollutant molecules (polychlorinated dibenzo-p-dioxins, polychlorinated dibenzofurans, and polychlorinated biphenyls) in Gold Rush, Black Beauty, and Patty Green zucchini subspecies. Because this accumulation process seems not to be a molecule-site equilibrium but a partition between two phases, and because there are early data showing that the logarithm of the octanol-water partition coefficient (a representation of lipophilicity) can be represented by the atomic indices used here [85–88], we conjecture that the method presented above can give better results than the Hansch or Hammett approaches.

**Figure 1:**
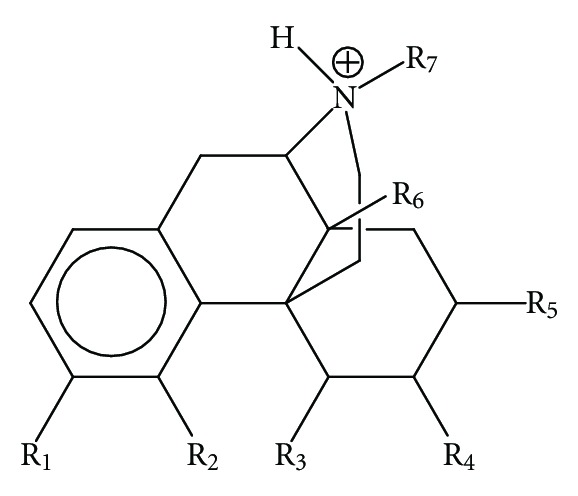
General formula of the molecules employed in this study.

The molecules selected for this study are shown in Figure 1 and Table 1, together with their corresponding experimental opioid receptor binding affinities [89]. 

**Figure 2:**
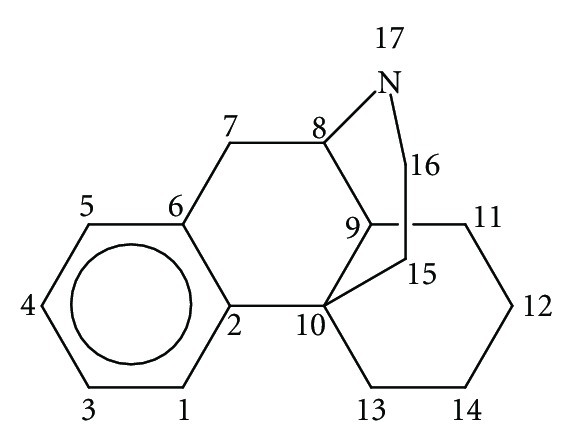
Common skeleton with atom numbering.

Our experience has shown that many times one or more of the components of (15) may be constant or negligible, but there is no *a priori* way to say with certainty which of these components can be eliminated in a specific study. On the other hand, regarding the drug atoms interacting with the receptor, the *common skeleton hypothesis* has given good results. This hypothesis states that there is a certain group of atoms, common to all molecules analyzed (called the common skeleton), that accounts for almost all the binding to the receptor. The action of the substituents consists in modifying the electronic structure of this skeleton (and influencing the correct alignment of the drug through the orientational parameters). For the case studied here the common skeleton is shown in Figure 2.

The obtention of the numerical values for the reactivity indices can be achieved at the semiempirical, HF or DFT levels of the theory. In our previous studies we have employed the semiempirical CNDO/2 and ZINDO/1 methods, *ab initio* HF and DFT methodologies. CNDO/2, HF, and DFT methods presented problems for the calculation of *S* ^*N*^ (see (8)); CNDO/2, because the empty MO eigenvalues it provides are highly dependent on the conformation [90]. HF, DFT, and CNDO/2 methodologies provide empty MOs with negative and positive energy eigenvalues producing algebraic zeros around the Fermi Level and leading to bad results for the total NSD. The ZINDO/1 method is designed in such a way that the empty MOs eigenvalues are always positive for neutral molecules, thus avoiding this algebraic problem. For the sake of comparison we carried out the calculations at the semiempirical ZINDO/1 and DFT B3LYP/6-31G(d,p) levels. The molecules were analyzed in their protonated form. Full geometry optimization was carried out (with OPLS for ZINDO/1 and B3LYP/6-31G(d,p) for DFT). ZINDO/1 calculations were performed with the Hyperchem software [91] and DFT ones with the Gaussian package [92]. With software written in our laboratory all the necessary information was extracted from the above commercial software and the local atomic reactivity indices were calculated. For the calculation of the local atomic reactivity indices all electron populations lesser or equal to 0.01 e were considered as zero. In the case of the DFT calculations, negative electron populations arising from Mulliken Population Analysis were corrected according to a recently proposed method [93]. We need to stress that, as we have shown during the last few years, the correct choice of more “primitive” methods (Extended Hückel Theory) can be very helpful in interpreting experimental results [[94–96] and references therein]. Linear multiple regression analysis was carried out with Statistica software [97]. The dependent variable is log IC_50_ and the independent variables are the set of local atomic reactivity indices plus the orientational parameters of the substituents R_1_–R_8_ (see Figure 1).

**Figure 3:**
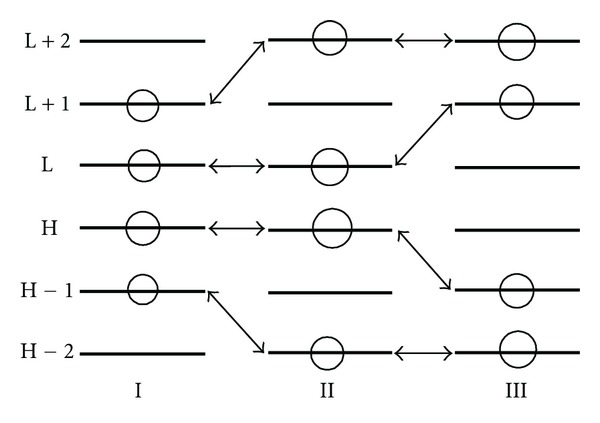
Atom *i* of molecules I, II, and III. Circles depict those MOs in which atom *i* has nonzero electron populations.

**Figure 4:**
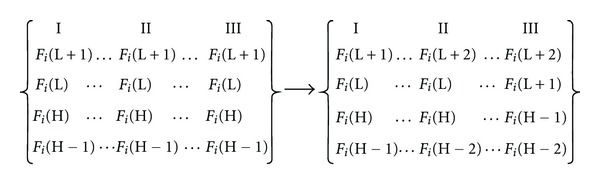
Left side: part of the original matrix data for atom *i* built from Figure 3. Right side: part of the final matrix data for atom *i* containing only nonzero values.

 A last word about variable ordering: Figure 3 shows an equivalent atom for molecules I, II, and III, each one with three “occupied” (H, H − 1, H − 2) and three “empty” (L, L + 1, L + 2) MOs. The circle in one MO means that the corresponding Fukui index of atom i has a non-zero value for this particular MO (e.g., in molecule I MOs H − 1, H, L, and L + 1). As the drug-receptor interaction is achieved through the existing (i.e., with nonzero electron populations) MOs of atom *i*, the data to be entered in the regression matrix must be modified as shown in Figure 4. The same holds for the orbital superdelocalizabilities. This must be done for all atoms entering in the multiple regression analysis.

## 3. Results

### 3.1. *μ*  Receptor Binding Affinity

#### 3.1.1. ZINDO/1 Results

The best equation obtained was

(17) log μ=7.18(±0.52)+0.20(±0.05)S3N(LUMO+1)−16.22(±1.13)η6+1.85(±0.37)Q13+0.81(±0.16)S13N(LUMO+2),

with *n* = 16, *R* = 0.98, *R* ^2^ = 0.97, *R*_adj_^2^ = 0.96, *F*(4,11) = 89.10 (*P* < 0.000001), outliers > 2*S* = 0, and SD = 0.16. Here *S*_3_^*N*^ (LUMO + 1) is the contribution of atom 3 to the second empty MO with non-zero electronic population, *η*_6_ is the hardness of atom 6, *Q*_13_ is the net charge of atom 13, and *S*_13_^*N*^ (LUMO + 2), its contribution to the third empty MO with non-zero electronic population. Tables 2 and 3 show, respectively, the beta coefficients, the results of the *t*-test for significance of coefficients, and the matrix of squared correlation coefficients for the variables appearing in (17).

**Figure 5:**
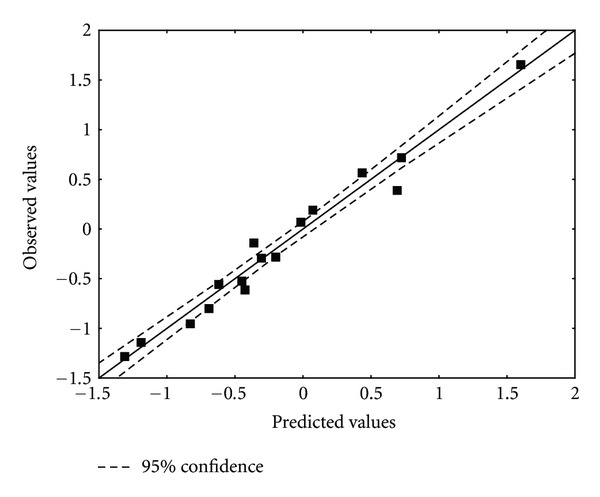
Observed and ZINDO/1 calculated values (17) of log(*μ*). Dashed lines denote the 95% confidence interval.

Table 4 shows the experimental and calculated values for log(*μ*).  Figure 5 displays calculated versus observed values. 

#### 3.1.2. B3LYP/6-31G(d,p) Results

 For log(*μ*) receptor binding affinity the best equation obtained is

**Figure 6:**
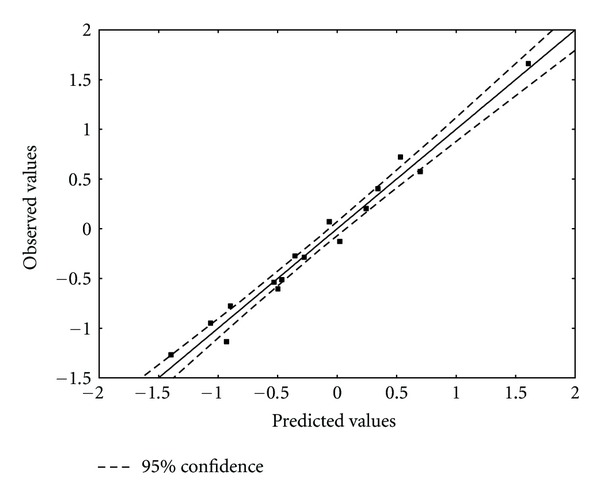
Observed and DFT calculated values (18) of log(*μ*). Dashed lines denote the 95% confidence interval.

(18) log μ=−15.91(±0.99)+5.50(±0.41)Q15max+49.75(±4.71)η5−29.43(±2.92)F11(LUMO)+10.11(±2.18)ω13,

with *n* = 16, *R* = 0.99, *R* ^2^ = 0.98, *R*_adj_^2^ = 0.97, *F*(4,11) = 113.62 (*P* < 0.00001), outliers > 2*S* = 0, and SD = 0.14. Here *Q*_15_^max^  is the maximal amount of electronic charge that atom 15 may accept, *η*_5_ is the hardness of atom 5, *F*_11_ (LUMO) is the Fukui index of atom 11 at the first empty MO with nonzero electronic population and *ω*_13_ is the electrophilic index of atom 13. Tables 5 and 6 show, respectively, the beta coefficients, the results of the *t*-test for significance of coefficients and the matrix of squared correlation coefficients for the variables appearing in (18). Table 4 shows the experimental and calculated values for log(*μ*).  Figure 6 displays calculated versus observed values.

### 3.2. *δ* Receptor Binding Affinity

#### 3.2.1. ZINDO/1 Results

 When the best statistical equation was obtained, it was observed that in the case of molecule 6 the corresponding standard residual fell outside the ±2 sigma limit (2.18). When a new linear multiple regression analysis was carried out without molecule 6, the standard residual for molecule 10 fell outside the ±2 sigma limit (2.30). As our interest is to compare results for the whole set, no attempt was made to get equations for smaller sets. We consider that the variation of the actual ZINDO/1 variables is not able to account for the variation of log(*δ*).

#### 3.2.2. B3LYP/6-31G(d,p) Results

 For the *δ* receptor binding affinity the best equation obtained is

**Figure 7:**
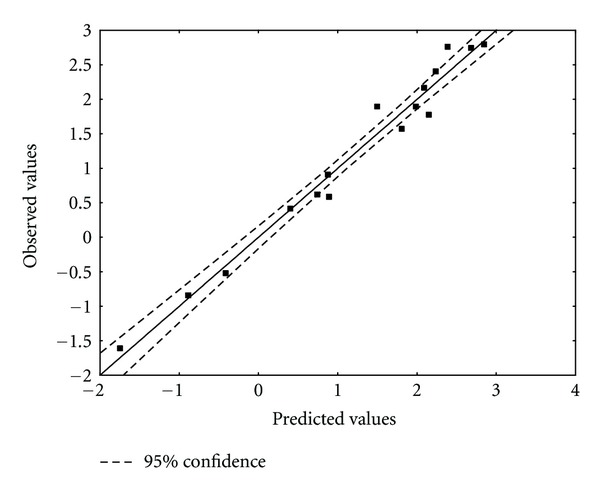
Observed and DFT calculated values (19) of log(*δ*). Dashed lines denote the 95% confidence interval.

(19) log δ=56.07(±3.18)+7.21(±0.41)S12E−6.82(±1.48)F13(HOMO)−0.19(±0.004)×S8N(LUMO+1)−0.78(±0.21)S10N(LUMO+1),

with *n* = 16, *R* = 0.99, *R* ^2^ = 0.98, *R*_adj_^2^ = 0.97, *F*(4,11) = 107.48 (*P* < 0.000001), outliers > 2*S* = 0 and SD = 0.25. Here *S*_12_^*E*^ is the total atomic electrophilic superdelocalizability of atom 12, *F*_13_ (HOMO) is the Fukui index of atom 13 at the highest occupied MO with non-zero electronic population, *S*_8_^*N*^ (LUMO + 1) is the orbital nucleophilic superdelocalizability of atom 8 at the second empty MO with non-zero electronic population, and *S*_10_^*N*^ (LUMO + 1) is the orbital nucleophilic superdelocalizability of atom 10 at the second empty MO with non-zero electronic population. Tables 7 and 8 show, respectively, the beta coefficients, the results of the *t*-test for significance of coefficients, and the matrix of squared correlation coefficients for the variables appearing in (19).  Table 9 shows the experimental and calculated values for log(*δ*).  Figure 7 displays calculated versus observed values.

### 3.3. *κ*  Receptor Binding Affinity Results

#### 3.3.1. ZINDO/1 Results

The best statistical equation obtained is

**Figure 8:**
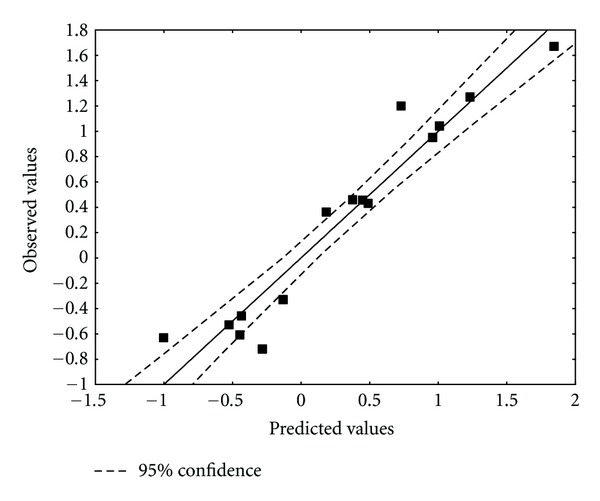
Observed versus ZINDO/1 calculated values (20) of log(*κ*). Dashed lines denote the 95% confidence interval.

(20) log κ=−8.33(±1.36)+0.71(±0.18)S7N(LUMO+1)+0.60(±0.14)S7N+41.66(±9.10)×F16(HOMO−1)+1.36(±0.45)×S11N(LUMO+1)+11.56(±4.02)×S11E(HOMO),

with *n* = 16, *R* = 0.96, *R* ^2^ = 0.92, *R*_adj_^2^ = 0.89, *F*(5,10) = 24.20 (*P* < 0.00003), outliers > 2*S* = 0, and SD = 0.27. Here S_7_^*N*^ (LUMO + 1) is the orbital nucleophilic superdelocalizability of atom 7 at the second empty MO with non-zero electronic population, *S*_7_^*N*^ is the total atomic nucleophilic superdelocalizability of atom 7, *F*_16_ (HOMO − 1) is the Fukui index of atom 16 at the second occupied MO with non-zero electronic population, *S*_11_^*N*^ (LUMO + 1) is the orbital nucleophilic superdelocalizability of atom 11 at the second empty MO with non-zero electronic population, and *S*_11_^*E*^ (HOMO) is the orbital electrophilic superdelocalizability of atom 11 at the highest occupied MO with non-zero electronic population. Tables 10 and 11 show, respectively, the beta coefficients, the results of the *t*-test for significance of coefficients and the matrix of squared correlation coefficients for the variables appearing in (20).  Table 12 shows the experimental and calculated values for log(*κ*).  Figure 8 displays calculated versus observed values. 

#### 3.3.2. B3LYP/6-31G(d,p) Results

**Figure 9:**
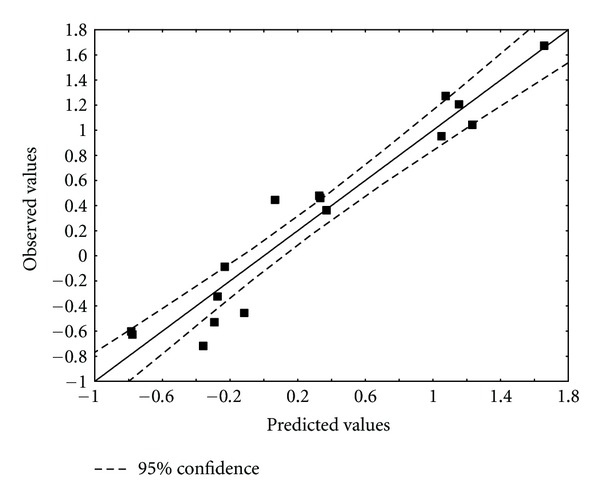
Observed versus DFT calculated values (21) of log(*κ*). Dashed lines denote the 95% confidence interval.

 For the *κ* receptor binding affinity the best equation obtained is:

(21) log κ=−3.22(±0.71)+27.44(±6.61)ω15+0.008(±0.001)S11N−1.46(±0.32)×S1E(HOMO−1)−16.55(±4.56)F11(LUMO)

with *n* = 16, *R* = 0.96, *R* ^2^ = 0.93, *R*_adj_^2^ = 0.91, *F*(4,11) = 36.78 (*P* < 0.00001), outliers > 2*S* = 0 and SD = 0.24. Here *ω*_15_ is the is the electrophilic index of atom 15, *S*_11_^*N*^ is the total atomic nucleophilic superdelocalizability of atom 11, *S*_1_^*E*^ (HOMO − 1) is the orbital electrophilic superdelocalizability of atom 1 at the second highest occupied MO with non-zero electronic population and *F*_11_ (LUMO) is the Fukui index of atom 11 at the first empty MO with non-zero electronic population. Tables 13 and 14 show, respectively, the beta coefficients, the results of the *t*-test for significance of coefficients, and the matrix of squared correlation coefficients for the variables appearing in (21).  Table 12 shows the experimental and calculated values for log(*κ*).  Figure 9 displays the calculated versus observed values.

## 4. Discussion

### 4.1. *μ*  Receptor Results

 The associated indices of (17) (ZINDO/1 results) and (18) (DFT results) show that both equations are statistically significant. Nevertheless, the results of the *t*-test for significance of the coefficients indicate that the DFT results are better. We note that DFT calculations provide a better representation of the electronic structure of molecules. Our previous studies were done at the CNDO/2 and ZINDO/1 levels of theory [29–31]. We cannot compare them with none of the results obtained here because the variable ordering, in the sense of Figures 3 and 4, was not carried out. We shall therefore only discuss the ZINDO/1 results for *μ* binding as an example of analysis.

**Figure 10:**
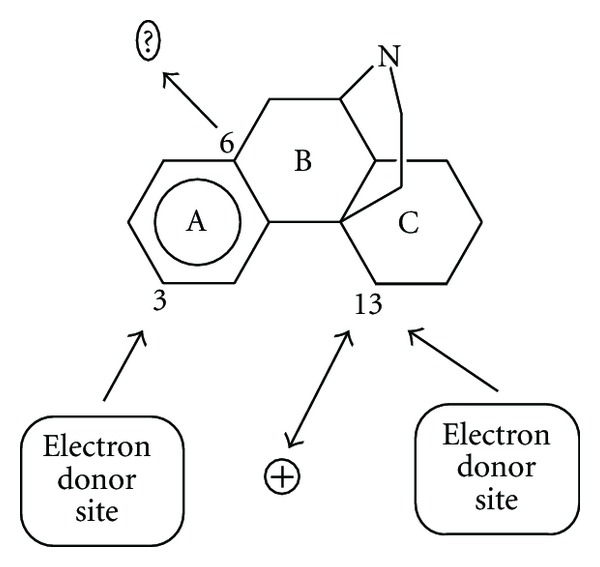
*μ* interaction pharmacophore from the ZINDO/1 results.

The ZINDO/1 results show that the variation of log(*μ*) is associated with the variation of several local atomic reactivity indices located at atoms 3, 6, and 13 of the common skeleton (see Figure 2).  Table 2 shows that the most important variable is the hardness (*η*) of atom 6. As *η* is positive, optimal binding is therefore associated with a high value of *η*_6_, indicating that atom 6 resists exchanging electrons with the environment (the receptor) and that it is a bad electrophile. At this moment it is not clear to us what the exact nature is of the region of the receptor near this atom. The appearance of *S*_3_^*N*^ (LUMO + 1) indicates that atom 3 faces an electron donor center of the *μ* receptor, probably a MO with *π* character. The appearance of the two variables for atom 13 is challenging.  Table 3 shows that they are not correlated and the beta values of Table 2 indicate that their relative effects on log(*μ*) are similar. On one hand *S*_13_^*N*^ (LUMO + 2) suggests that atom 13 receives charge from an occupied *π* MO of the receptor. On the other, the optimal value of *Q*_13_ should be negative. A possibility is that atom 13 binds the *μ* receptor in such a way that one side faces the receptor MO while another side interacts with a positively charged site.  Figure 10 summarizes these ideas.

**Figure 11:**
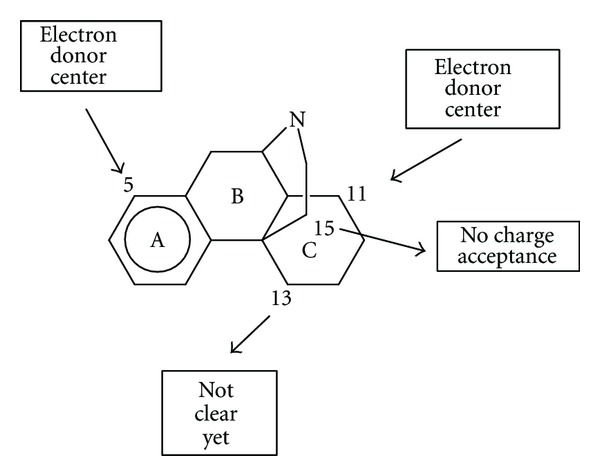
*μ* interaction pharmacophore from the DFT results.

In the case of the DFT results (18) we find that the variation of the affinity is associated with the hardness of atom 5, with an interaction of an empty MO in atom 11 with an electron donor site in the receptor, with the electrophilicity of atom 13, and with the maximal amount of electron charge that atom 15 may accept as an electrophile (see Figure 2 for atom numbering). The most important index is the maximal amount of electron charge that atom 15 may accept (see Table 5). The value for the hardness of atom 5 should be low, implying that this atom should be a good electrophile. Atom 15 needs to have a low capacity to accept charge, which is expected for a CH_2_ carbon atom. Atom 11 interacts through one of its empty MOs with an electron donor center of the receptor. Atom 13 should have low electrophilicity. As this index includes the tendency of atom 13 to receive extra electric charge together with its resistance to exchange charge with the medium we may say, in a first approach, that in this atom the resistance to exchange charge with the medium is higher than its tendency to receive charge. All these facts are summarized in Figure 11. It is interesting to note that *μ* binding seems to be charge and orbital controlled [43–45].

### 4.2. *δ*  Receptor Results

**Figure 12:**
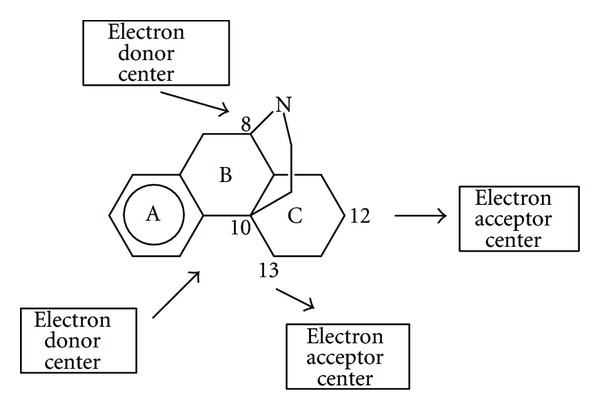
*δ* interaction pharmacophore from the DFT results.

 The associated indices of (19) (DFT results) indicate that this equation is statistically significant. The most important index is the total atomic electrophilic superdelocalizability of atom 12 (see Table 7). We may see that atoms 8 and 10 act as electron acceptors through the second empty MO with non-zero (virtual) electron populations. Atoms 12 and 13 act as electron donors. Contrary to the DFT results for *μ* binding, *δ* binding involves Molecular Orbitals other than the HOMO and LUMO. Perhaps this is one of the mechanisms regulating not only affinity, but also selectivity. The proposed *δ* interaction pharmacophore is shown in Figure 12.

### 4.3. *κ*  Receptor Results

**Figure 13:**
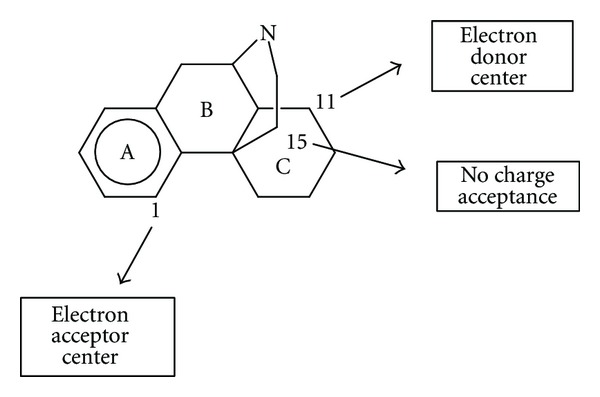
*κ*interaction pharmacophore from the DFT results.

 The associated indices of (21) (DFT results) indicate that this equation is statistically significant. Here, *ω*_15_, *S*_11_^*N*^ and *S*_1_^*E*^ (HOMO − 1) have the same importance (see Table 13). Atom 15 should have low electrophilicity implying, as in the case of *μ* binding, that in this atom the resistance to exchange charge with the medium is higher than its tendency to receive charge. *S*_11_^*N*^ and *F*_11_  (LUMO) indicate that atom 11 transfers charge to an electron-deficient center in the receptor. These two variables are not correlated (see Table 14). Atom 1 also transfers charge to an electron-deficient center.  Figure 13 summarizes these ideas.

The appearance of local reactivity indices belonging to atom 15 is interesting because they can give an account of the difference in receptor affinity between (+) and (−) isomers. This fact needs to be supported by more studies like this one.

Figures 10–13 are planar representations of tridimensional interaction pharmacophores. What must be done to improve our knowledge? We need to apply this method to more experimental data. In this sense, as the IC_50_ values must be measured under almost the same experimental conditions, we are limited to papers reporting a large number of experimental results. IC_50_ values coming from different sources cannot be merged into one set [98]. Results with more extended sets of molecules may help to identify the exact nature of the receptor sites with which opiate molecules interact. Another line of research is to test this model for agonist and antagonist properties to see if it is possible to build the corresponding pharmacophores. The study of enkephalin analogues seems to be possible because there are several articles reporting binding data and pharmacological profiles for large sets of molecules.

 The main conclusions of this work are as follows 1. some of the new local atomic reactivity indices of the extended model appear in the resulting statistical equations; 2. the interaction pharmacophores are different for *μ*, *δ*, and *κ* receptors and this difference may account for selectivity; 3. *μ* and *κ* interaction pharmacophores seem to share common features.

## Figures and Tables

**Table 1:** Molecules and their experimental opioid receptor binding affinities^a,b^.

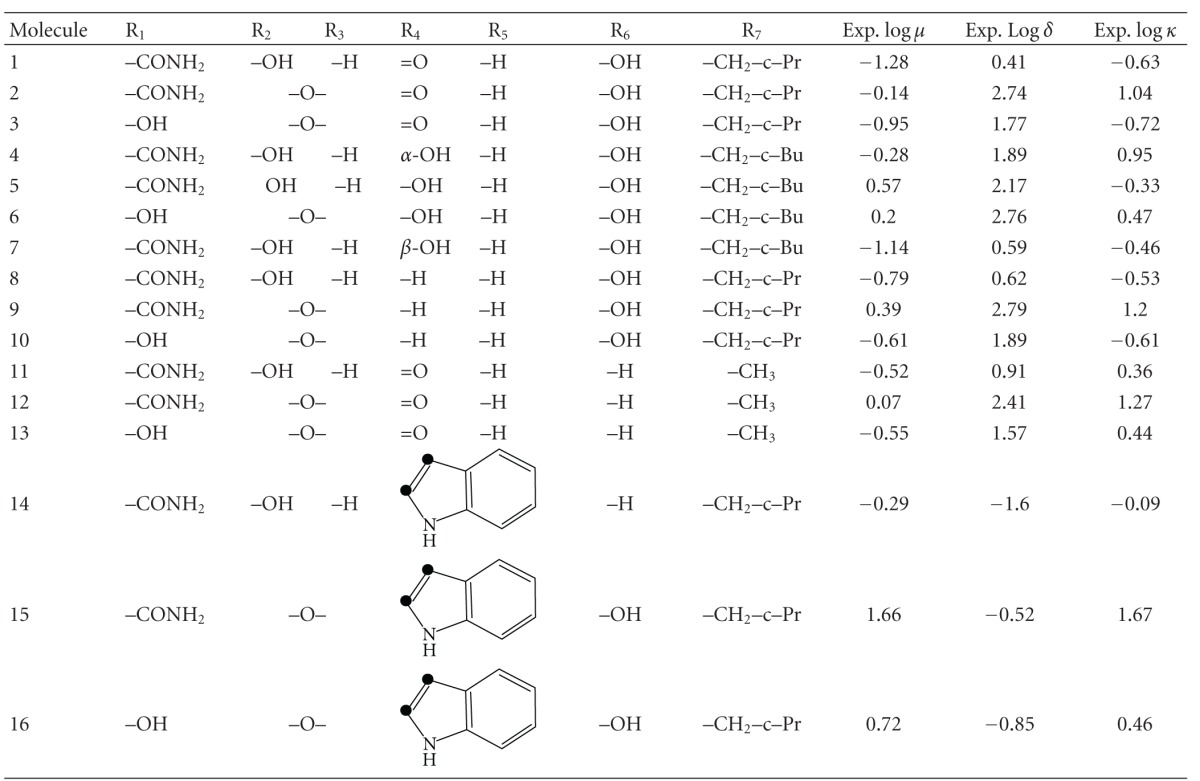

^a^From [89]. ^b^In molecules 15–17 the carbon atoms marked with black dots belong to the common skeleton.

**Table 2:** Beta coefficients and *t*-test for significance of coefficients in (17).

Variable	Beta coefficient	*t*	*P*
*S*_3_^*N*^(LUMO + 1)	0.24	3.93	<0.0023
*η*_6_	−0.84	−14.39	<0.000001
*Q*_13_	0.32	4.94	<0.0004
*S*_13_^*N*^(LUMO + 2)	0.31	5.16	<0.0003

**Table 3:** Squared correlation coefficients for the variables appearing in (17).

	*S*_13_^*N*^(LUMO + 1)	*η*_6_	*Q*_13_	*S*_13_^*N*^(LUMO + 2)
*S*_3_^*N*^(LUMO + 1)	1.0			
*η*_6_	0.14	1.0		
*Q*_13_	0.005	0.026	1.0	
*S*_13_^*N*^(LUMO + 2)	0.036	0.002	0.19	1.0

**Table 4:** Observed and calculated log(mu) values.

Molecule	Exp log(*μ*)^a^	Calculated log(*μ*)^b^ZINDO/1	Calculated log(*μ*)^c^DFT
1	−1.28	−1.32	−1.39
2	−0.14	−0.37	0.03
3	−0.95	−0.83	−1.06
4	−0.28	−0.21	−0.35
5	0.57	0.43	0.70
6	0.2	0.07	0.25
7	−1.14	−1.19	−0.92
8	−0.79	−0.70	−0.89
9	0.39	0.69	0.35
10	−0.61	−0.43	−0.49
11	−0.52	−0.45	−0.46
12	0.07	−0.02	−0.06
13	−0.55	−0.63	−0.52
14	−0.29	−0.31	−0.28
15	1.66	1.60	1.61
16	0.72	0.72	0.54

^a^Reference [89]. ^b^With (17). ^c^With (18).

**Table 5:** Beta coefficients and *t*-test for significance of coefficients in (18).

Variable	Beta coefficient	*t*	*P *
*Q*_15_^max^	0.73	13.19	<0.000001
*η*_5_	0.51	10.56	<0.000001
*F*_11_(LUMO)	−0.54	−10.06	<0.000001
*ω*_13_	0.29	4.65	<0.0007

**Table 6:** Squared correlation coefficients for the variables appearing in (18).

	*η*_5_	*F*_11_(LUMO)	*ω*_13_	*Q*_15_^max^
*η*_5_	1.0			
*F*_11_(LUMO)	0.004	1.0		
*ω*_13_	0.05	0.20	1.0	
*Q*_15_^max^	0.0006	0.02	0.25	1.0

**Table 7:** Beta coefficients and *t*-test for significance of coefficients in (19).

Variable	Beta coefficient	*t*	*P*
*S*_12_^*E*^	0.90	17.53	<0.0000001
*F*_13_(HOMO)	−0.24	−4.63	<0.0007
*S*_8_^*N*^(LUMO + 1)	−0.29	−5.70	<0.0001
*S*_10_^*N*^(LUMO + 1)	−0.18	−3.66	<0.0038

**Table 8:** Squared correlation coefficients for the variables appearing in (19).

	*F*_8_(HOMO − 2)	*F*_10_(HOMO − 2)	*S*_11_^*E*^	*μ*_12_
*F*_8_(HOMO − 2)	1.0			
*F*_10_(HOMO−2)	0.04	1.0		
*S*_11_^*E*^	0.06	0.03	1.0	
*μ*_12_	0.02	0.04	0.06	1.0

**Table 9:** Observed and calculated log(delta) values.

Molecule	Exp log(delta)^a^	Calculated log(delta)^b^ DFT
1	0.41	0.40
2	2.74	2.68
3	1.77	2.16
4	1.89	1.50
5	2.17	2.09
6	2.76	2.39
7	0.59	0.89
8	0.62	0.74
9	2.79	2.85
10	1.89	1.98
11	0.91	0.88
12	2.41	2.24
13	1.57	1.81
14	−1.6	−1.75
15	−0.52	−0.41
16	−0.85	−0.89

^a^Reference [89]. ^b^With (19).

**Table 10:** Beta coefficients and *t*-test for significance of coefficients in (20).

Variable	Beta coefficient	*t*	*P*
*S*_7_^*N*^(LUMO + 1)	0.45	3.96	<0.0027
*S*_7_^*N*^	0.65	5.52	<0.0003
*F*_16_(HOMO − 1)	0.45	4.55	<0.0011
*S*_11_^*N*^(LUMO + 1)	0.29	3.01	<0.0130
*S*_11_^*N*^(HOMO)	0.29	2.87	<0.0166

**Table 11:** Squared correlation coefficients for the variables appearing in (20).

	*S*_7_^*N*^	*S*_7_^*N*^(LUMO + 1)	*S*_11_^*E*^(HOMO)	*S*_11_^*N*^(LUMO + 1)	*F*_16_(HOMO − 1)
*S*_7_^*N*^	1.0				
*S*_7_^*N*^(LUMO + 1)	0.25	1.0			
*S*_11_^*E*^(HOMO)	0.03	0.06	1.0		
*S*_11_^*N*^(LUMO + 1)	0.0004	0.02	0.05	1.0	
*F*_16_(HOMO − 1)	0.16	0.04	0.008	0.04	1.0

**Table 12:** Observed and calculated log(*κ*) values.

Molecule	Exp log(*κ*)^a^	Calculated log(*κ*)^b^ ZINDO/1	Calculated log(*κ*)^c^ DFT
1	−0.63	−1.00	−0.78
2	1.04	1.01	1.23
3	−0.72	−0.28	−0.36
4	0.95	0.96	1.06
5	−0.33	−0.13	−0.27
6	0.47	0.45	0.33
7	−0.46	−0.43	−0.11
8	−0.53	−0.52	−0.29
9	1.2	0.73	1.16
10	−0.61	−0.44	−0.78
11	0.36	0.19	0.37
12	1.27	1.23	1.08
13	0.44	0.48	0.07
14	−0.09	0.03	−0.23
15	1.67	1.85	1.66
16	0.46	0.38	0.34

^a^Reference [89]. ^b^With (20). ^c^With (21).

**Table 13:** Beta coefficients and *t*-test for significance of coefficients in (21).

Variable	Beta coefficient	*t*	*P*
*ω*_15_	0.44	4.15	<0.0008
*S*_11_^*N*^	0.51	6.34	<0.002
*S*_1_^*E*^(HOMO − 1)	−0.47	−4.51	<0.00006
*F*_11_(LUMO)	−0.30	3.63	<0.004

**Table 14:** Squared correlation coefficients for the variables appearing in (21).

	*S*_1_^*E*^(HOMO − 1)	*S*_11_^*N*^	*F*_11_(LUMO)	*ω*_15_
*S*_1_^*E*^(HOMO − 1)	1.0			
*S*_11_^*N*^	0.008	1.0		
*F*_11_(LUMO)	0.02	0.0004	1.0	
*ω*_15_	0.41	0.004	0.05	1.0

## Aknowledgments

Prof. Dr. Bruce K. Cassels is gratefully acknowledged for very helpful comments. This work received financial help from AOG.
